# Reticular fibre structure in the differential diagnosis of parathyroid neoplasms

**DOI:** 10.1186/s13000-023-01368-y

**Published:** 2023-07-04

**Authors:** Xiumei Hu, Shurong He, Xingran Jiang, Ping Wei, Xiang Zhou, Zhongyue Shi, Xue Li, Jun Lu, Hongying Zhao, Bojun Wei, Mulan Jin

**Affiliations:** 1 Department of Pathology, Beijing Chaoyang Hospital, Capital Medical University, Beijing, 100020 China; 2 Department of Pathology, Beijing Hospital, National Center of Gerontology, Institute of Geriatric Medicine, Chinese Academy of Medical Sciences, Beijing, 100730 China; 3 Department of Thyroid and Neck Surgery, Beijing Chaoyang Hospital, Capital Medical University, Beijing, 100020 China

**Keywords:** Parathyroid neoplasm, Reticular fibre structure, Traversable field line, Diagnosis

## Abstract

**Background:**

To investigate the characteristics of reticular fibre structure (RFS) in parathyroid adenoma (PTA), atypical parathyroid tumour (APT), and parathyroid carcinoma (PTC), and to assess its value as a diagnostic indicator.

**Methods:**

Clinical data and pathological specimens of patients with PTA, APT or PTC were collected. Reticular fibre staining was performed to observe the characteristics of RFS. This study evaluated the incidence of RFS destruction in parathyroid tumours, compared RFS destruction between primary PTC and recurrent and metastatic PTC, and explored the association between RFS destruction and clinicopathological features of APT and primary PTC.

**Results:**

Reticular fibre staining was performed in 50 patients with PTA, 25 patients with APT, and 36 patients with PTC. In PTA cases, a delicate RFS was observed. In both the APT and PTC groups, incomplete RFS areas were observed. The incidence of RFS destruction was different among the PTA, APT, and PTC groups (P < 0.001, χ^2^-test), at 0% (0/50), 44% (11/25), and 86% (31/36), respectively. When differentiating PTC from APT, the sensitivity and specificity of RFS destruction were 81% and 56%, respectively. The incidence of RFS destruction was 73% (8/11) in the primary PTC group and 92% (23/25) in the recurrent and metastatic PTC groups. In both the APT group and primary PTC group, no correlation was found between RFS destruction and clinicopathological features.

**Conclusion:**

RFS destruction may indicate that parathyroid tumours have unfavourable biological behaviours.Reticular fibre staining may be a valuable tool for improving the diagnostic accuracy in parathyroid tumours.

## Background

The spectrum of parathyroid neoplasms encompasses parathyroid adenomas (PTA), atypical parathyroid tumours (APT), and parathyroid carcinomas (PTC) [1]. APT has been newly described in the 2022 World Health Organization (WHO) classification system. As a tumour with uncertain malignant potential, although most APTs do not recur after resection, similar to PTAs, recurrence and metastases have been reported in some cases. Therefore, the APTs usually require long-term follow-up [2]. For patients with PTC, radical surgery, including en bloc resection of the tumour, ipsilateral thyroid tissue, and the surrounding adjacent tissues, is recommended. Due to these significant differences in the biological behaviour and treatment options, accurate differentiation of these tumours is crucial. According to the WHO criteria, PTC diagnosis should be based on the presentation of one of the following clinicopathological features: lymphovascular invasion, perineural invasion, direct spread into adjacent structures, or distant metastasis [1]. APT is a parathyroid neoplasm that demonstrates atypical cytological and architectural features but lacks unequivocal capsular, vascular, perineural invasion, or invasion into adjacent structures or metastases [1]. However, the complexity of their morphological structure, subjectivity of pathological diagnosis, and inconsistency between observers contribute to the uncertainty of the pathological diagnosis, thereby resulting in poor treatment effects and inadequate follow-up in the case of parathyroid tumours. Consequently, auxiliary diagnostic methods are necessary to improve the diagnosis and risk evaluation of these tumours.

Recently, antibodies to proteins such as parafibromin, Ki67, and P27 have been developed for differential diagnosis. However, in most cases, the application of this method is limited by problems such as diagnostic overlap, technical difficulties, inter-observer variability, and inconsistencies in the evaluation criteria [3].

Reticular fibres are mainly composed of type III collagen and are widely distributed throughout the body. Under haematoxylin and eosin (HE) staining, reticular fibres are slender and difficult to observe. Furthermore, there is a layer of acidic proteoglycan on the surface of reticular fibres. After being dipped in silver ammonia solution, it can be reduced by formaldehyde to make it black, so that the reticular fibres in the tissue can be clearly coloured. Reticular fibre staining is a fast, cheap, stable, and easily interpreted staining method that can reveal changes in the amount, thickness, density, fracture, collapse, and other morphological characteristics of these fibres. This technique can assist in displaying the reticular fibre structure (RFS) around endocrine gland cells and capillaries, which is important for assessing the nature, degree, development, and outcome of lesions. In the diagnosis of pituitary adenoma, hepatocellular carcinoma, and adrenocortical tumours, reticular fibre staining has been used to observe changes in RFS and to determine the nature of the tumour. Kuçabuk et al. first reported RFS of PTA. They reported that the main RFS in PTA can be defined as short, thick fibre, anastomotic fibre, and nodular fibre types [4]. The short, thick reticular fibres are associated with PTA and hyperplasia, whereas the nodular type supports PTA [4]. However, APT and PTC were not investigated in their study.

Therefore, in the present study, we investigated the characteristics of RFS in PTA, APT, and PTC and assessed the value of RFS as a diagnostic indicator for parathyroid neoplasms.

## Methods

### Study inclusion criteria

The study was approved by the ethical committee of Beijing Chaoyang Hospital, Capital Medical University. We searched the computerized database of the Department of Pathology, Beijing Chaoyang Hospital, Capital Medical University, from January 2018 to August 2022, for all cases that were diagnosed with APT or PTC. The inclusion criteria for the APT and PTC group were as follows: (1) availability of complete clinical data, (2) absence of adjuvant therapy or biopsy before surgery, (3) availability of sufficient archived tissue for reticular fibre staining. In addition, 50 PTA cases diagnosed between January 2019 and December 2019 were selected as controls. The inclusion criteria for the PTA group were as follows: (1) no recurrence for 3 years after surgery; (2) the specimens were surgical tumour resection specimens; and (3) the postoperative pathological diagnosis was clear.

The clinical features of the patients were collected as follows: sex, age, tumour size, blood calcium, and blood parathyroid hormone (PTH) levels. The tumour size was measured along the maximum diameter of the tumour. The blood calcium or PTH level was defined as the highest level before the operation.

### Pathologic diagnosis

All patients underwent morphological review of all available HE-stained sections and were diagnosed by two endocrine pathologists (JML and HXM). Only cases that were approved by both pathologists were included. The patients were classified as having PTA, APT, or PTC using the criteria outlined in the 2022 WHO Classification of Endocrine Neoplasia. PTC diagnosis required a demonstration of unequivocal invasive growth with one of the following histological features: vascular invasion, lymphatic invasion, perineural invasion, adjacent anatomic structures invasion, and documentation of metastatic disease [1]. Patients with recurrence or metastasis were classified as the PTC group. APT was defined as a parathyroid neoplasm that demonstrated atypical cytological and architectural features that indicated PTC but lacked unequivocal capsular, vascular, or perineural invasion or invasion into adjacent structures or metastases [1]. In patients with APT and primary PTC, the presence of specific morphological features, including incomplete capsule invasion, fibre bands, apparent flake growth, large nucleoli, mitotic activity > 5/50 high-power fields (HPF), coagulation necrosis, and cytological atypia were also specifically investigated and recorded.

After observing all HE-stained sections, we prioritized sections with diffuse/nodular/trabecular growth patterns of tumour tissue for reticular fibre staining. If no such sections were available, we selected the section with the densest tumour cells.

### Reticular fibre staining method

Four-micron-thick sections were cut from all specimens and stained with a silver impregnation-based kit for reticulin staining (Artisan Link Pro, Agilent, Santa Clara, CA, USA; Ventana BenchMark Ultra, Ventana Medical Systems Inc., Oro Valley, AZ, USA). Staining was performed according to the manufacturer’s instructions.

### Interpretation of the reticular fibre staining results

After reticulin fibre staining, the RFS of whole glands was evaluated first with low magnification (40×, 100×), followed by high magnification (400×), using an OLYMPUS BX 53 microscope (Olympus Corp., Shinjuku, Japan).

### Definition of RFS destruction based on reticular fibre staining

This study adopted the traversable field method described by Jarzembowski et al. [5] for the diagnosis of pituitary adenoma, which refers to the pathway from any point on the periphery of a single field to a point on the opposite side of the field that does not encounter any stained fibres, using 10× objective lens magnification. We refer to this pathway as a traversable line. If a traversable line was observed, it represented RFS destruction. In some cases of PTC, fibrous tissue hyperplasia was present around nodular tumour cell nests. If a traversable line was present, it was considered to represent the destruction of the RFS in these cases, regardless of the nest size.

### Statistical analysis

IBM SPSS 27.0 (IBM SPSS Inc., Armonk, NY, USA), was used for statistical analyses. The χ^2^-test with Fisher’s exact test, as appropriate, was used to examine relationships between categorical variables. Statistical significance was set at P < 0.05.

## Results

### Clinicopathological findings

In total, 111 lesions from 106 patients were included in this study. Of the 111 lesions, 50 were PTA, 25 were APT, and 36 were PTC (including 11 primary, 19 recurrent, and six metastatic lesions). Of the 50 patients with PTA, 41 were women and 9 were men and their mean age was 42.7 years (range, 31–78 years). There were 28 men and 33 women among the APT and PTC cases and their mean age was 49.6 years (min: 19; max: 80 years). The clinical and demographic details of the APT and PTC patients, including tumour size, preoperative calcium and parathyroid hormone levels, and diagnosis, are presented in Table 1.

**Table 1:** Clinicopathologic data and RFS diagnosis of APT and PTC cases

Specimen	Patient	Sex	Age	Tumour size	Blood calcium	Blood PTH level	WHO 2022 diagnosis	RFS diagnosis
1	1	F	27	2	4.06	333	APT	D
2	2	M	69	1.4	2.86	130	APT	ND
3	3	F	53	UN	3.72	>1900	APT	ND
4	4	M	64	4	4.8	92.7	APT	D
5	5	F	57	1.6	2.88	26.13	APT	ND
6	6	M	36	4	3.53	985	APT	D
7	7	M	28	5	3.31	763	APT	D
8	8	M	51	1.1	2.73	407	APT	ND
9	9	F	55	1.4	2.73	178	APT	ND
10	10	M	19	2.6	2.76	935	APT	ND
11	11	F	55	2.5	2.96	321	APT	ND
12	12	F	80	2.1	3.55	400	APT	D
13	13	F	47	2	2.26	198	APT	ND
14	14	F	70	2.4	4.56	>3000	APT	ND
15	15	F	58	2.7	2.09	1046	APT	D
16	16	M	64	2.1	3.08	246	APT	D
17	17	F	72	1.5	2.68	195	APT	ND
18	18	F	35	1.7	2.84	149	APT	D
19	19	F	74	2.1	2.74	123	APT	ND
20	20	F	57	1.2	2.67	163	APT	ND
21	21	M	34	2.2	2.8	275	APT	ND
22	22	F	32	1.7	3	1075	APT	D
23	23	M	19	2.6	2.7	935	APT	D
24	24	F	47	1.9	2.44	988	APT	D
25	25	F	68	2.1	3	407	APT	ND
26	26	F	58	2.2	3.1	258	P-PTC	ND
27	27	F	56	1.6	3.23	204	P-PTC	ND
28	28	F	56	2.5	3.23	3521	P-PTC	D
29	29	F	72	1.5	2.98	285.9	P-PTC	D
30	30	M	51	2.9	2.98	349.8	P-PTC	D
31	31-A	M	44	10	4.08	149.6	P-PTC	D
32	31-B	M	44	10	4.08	149.6	M-PTC	D
33	32	M	59	2.2	2.27	UN	P-PTC	D
34	33	M	38	1.1	2.48	77.8	P-PTC	D
35	34	M	80	2.6	2.9	294	P-PTC	ND
36	35	M	74	2.9	2.8	139	P-PTC	D
37	36	M	53	3	3.5	1100	P-PTC	D
38	37	M	28	2	2.55	1156	M-PTC	D
39	38	M	61	2.5	4.1	1728	R-PTC	D
40	39-A	F	47	2	3.5	1092	M-PTC	D
41	39-B	F	47	2	3.5	1092	R-PTC	D
42	40	M	42	2.6	3.49	2334	R-PTC	D
43	41	F	29	2.5	5.84	1507	R-PTC	D
44	42	M	65	UN	UN	UN	R-PTC	D
45	43	F	38	2.5	3.81	953	R-PTC	D
46	44	F	31	1.3	3.21	540	R-PTC	D
47	45-A	M	45	3.2	3.67	1601	R-PTC	D
48	45-B	M	45	3.2	3.67	1601	R-PTC	D
49	45-C	M	45	3.2	3.67	1601	M-PTC	D
50	46	M	28	4	3.34	>2000	R-PTC	D
51	47	M	33	3.2	2.72	229	R-PTC	ND
52	48	M	53	UN	4	>1000	R-PTC	D
53	49	F	53	UN	UN	UN	R-PTC	D
54	50	M	37	UN	3.38	142	R-PTC	D
55	51	F	64	2.8	4.43	1081	R-PTC	D
56	52-A	M	49	3	4.2	728	R-PTC	D
57	52-B	M	49	3	4.2	728	M-PTC	D
58	53	M	31	UN	2.65	1170	M-PTC	ND
59	54	F	51	2.3	4.26	1389	R-PTC	D
60	55	M	31	1.3	4.2	1659	R-PTC	D
61	56	M	67	UN	UN	UN	R-PTC	D

Abbreviations: M, male; F, female; UN, unknown; RFS, reticular fibre structure; PTA, parathyroid adenoma; APT, atypical parathyroid tumour; PTC, parathyroid carcinoma; PTH, parathyroid hormone; P-PTC, primary PTC; R-PTC, recurrent PTC; M-PTC, metastatic PTC; D, destruction; ND, no destruction

#### Characteristics of RFS in parathyroid tumours

**Fig. 1:**
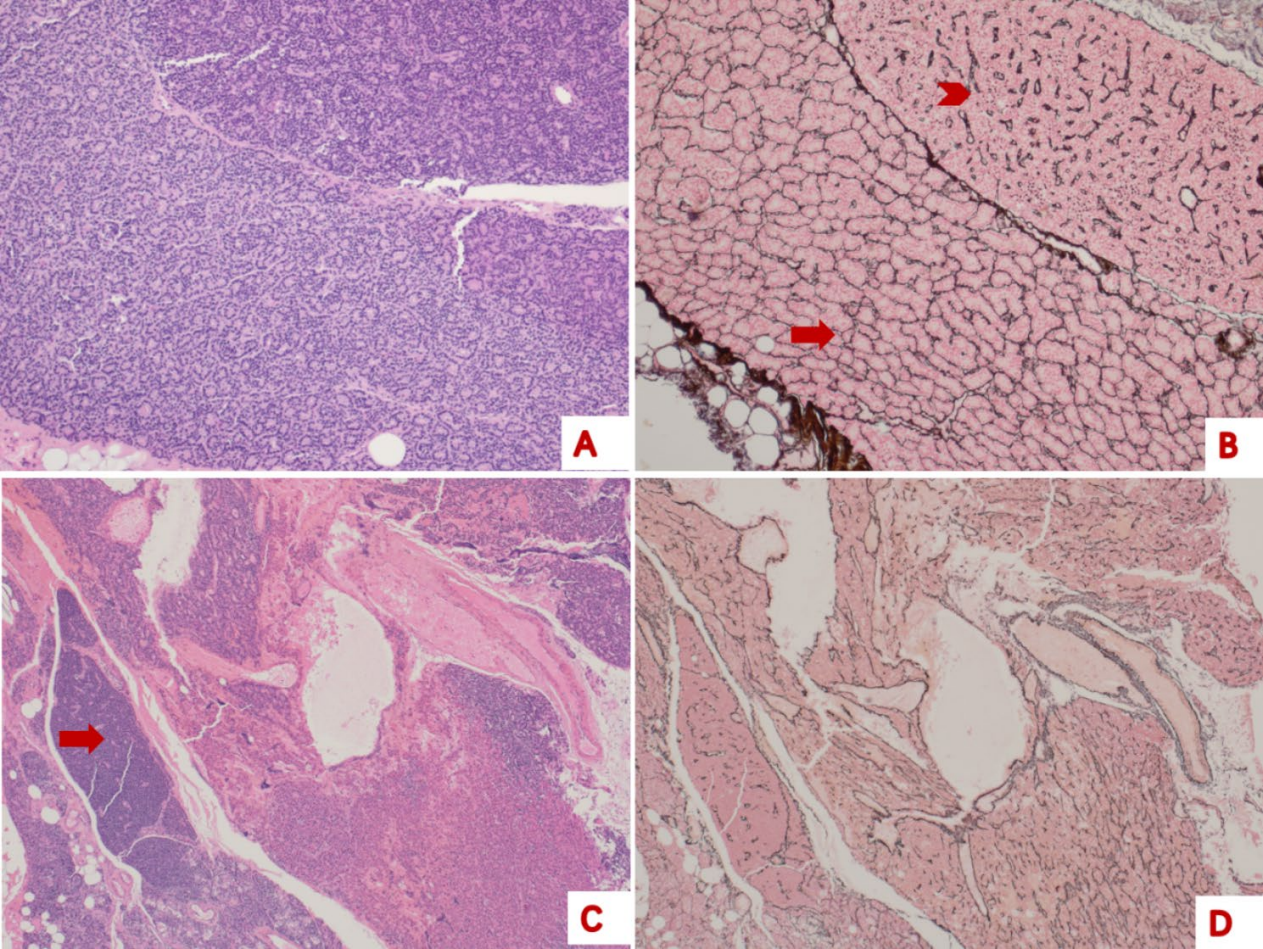
Representative histological features and reticular fibre staining of parathyroid adenoma **A**, parathyroid adenoma (haematoxylin eosin, 40×). **B**, reticular fibre staining showing the reticular structure (arrow) and irregular sinusoid structure (arrowhead) (40×). **C**, parathyroid adenoma, normal parathyroid tissue (arrow) can be seen around the parathyroid adenoma (haematoxylin eosin, 40×). **D**, reticular fibres are sparsely distributed in certain areas, reticular fibre structure destruction is absent (reticular fibre staining, 40×)

Reticular fibre staining showed the following RFS characteristics in PTA cases (Fig. 1). A network of reticular fibres surrounded the parathyroid cells in the pattern of nests or cords, and these fibres formed anastomoses with each other. The reticular structures could be large or small, and the main structure contained multiple structures. The reticular fibres were evenly distributed and uniform in thickness. The RFS was discontinuous in some areas where the reticular fibres were stained as irregular sinusoids, short and thick, or small tubes, but no traversable line was present. On the corresponding HE-stained sections, there was no evidence of gland structure destruction in the region.

**Fig. 2:**
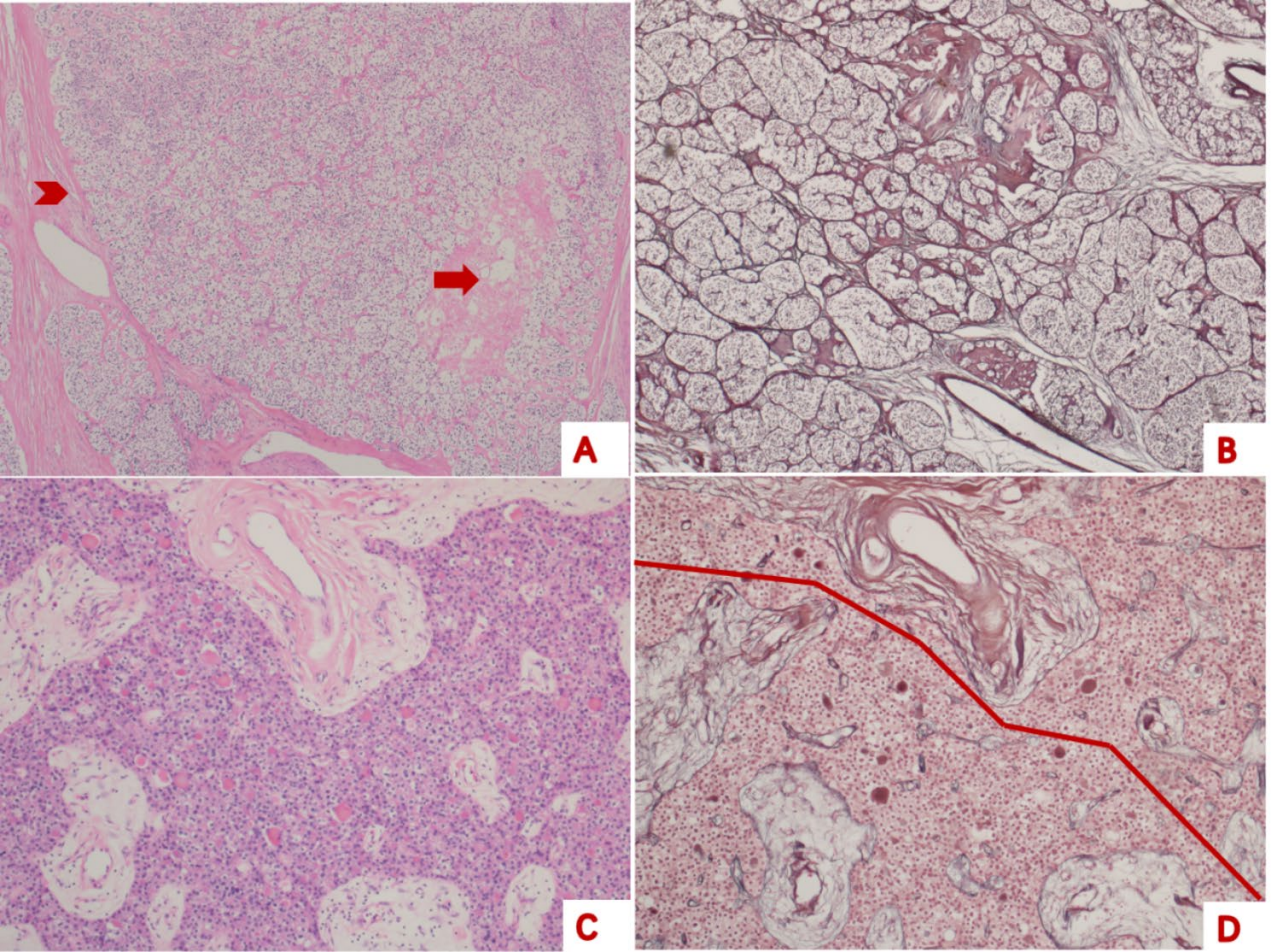
Representative histological features and reticular fibre staining of atypical parathyroid tumour **A**, the presence of coagulation necrosis (arrow) and fibre bands (arrowhead) supports the diagnosis of atypical parathyroid tumours (haematoxylin eosin, 40×). **B**, the anastomotic reticular fibre structure can be seen, the network structure varies in size, but no areas are destroyed (reticular fibre staining, 40×). **C**, the presence of fibre bands and obvious flake supports the diagnosis of atypical parathyroid tumours (haematoxylin eosin, 40×). **D**, a traversable field line can be seen, indicating the destruction of reticular fibre structure (reticular fibre staining, 40×)

Reticular fibre staining revealed that the RFS of APT primarily showed RFS destruction (Fig. 2). Destruction of the original nest-like or cord-like structure was noted, and the reticular fibres around the tumour cells showed an irregular shape or mosaic pattern. In some areas, reticular fibres were sparsely distributed and presented irregular sinusoids. The RFS structure was destroyed in some areas, and a traversable line could be seen, whereas a normal RFS was observed in the surrounding area.

**Fig. 3:**
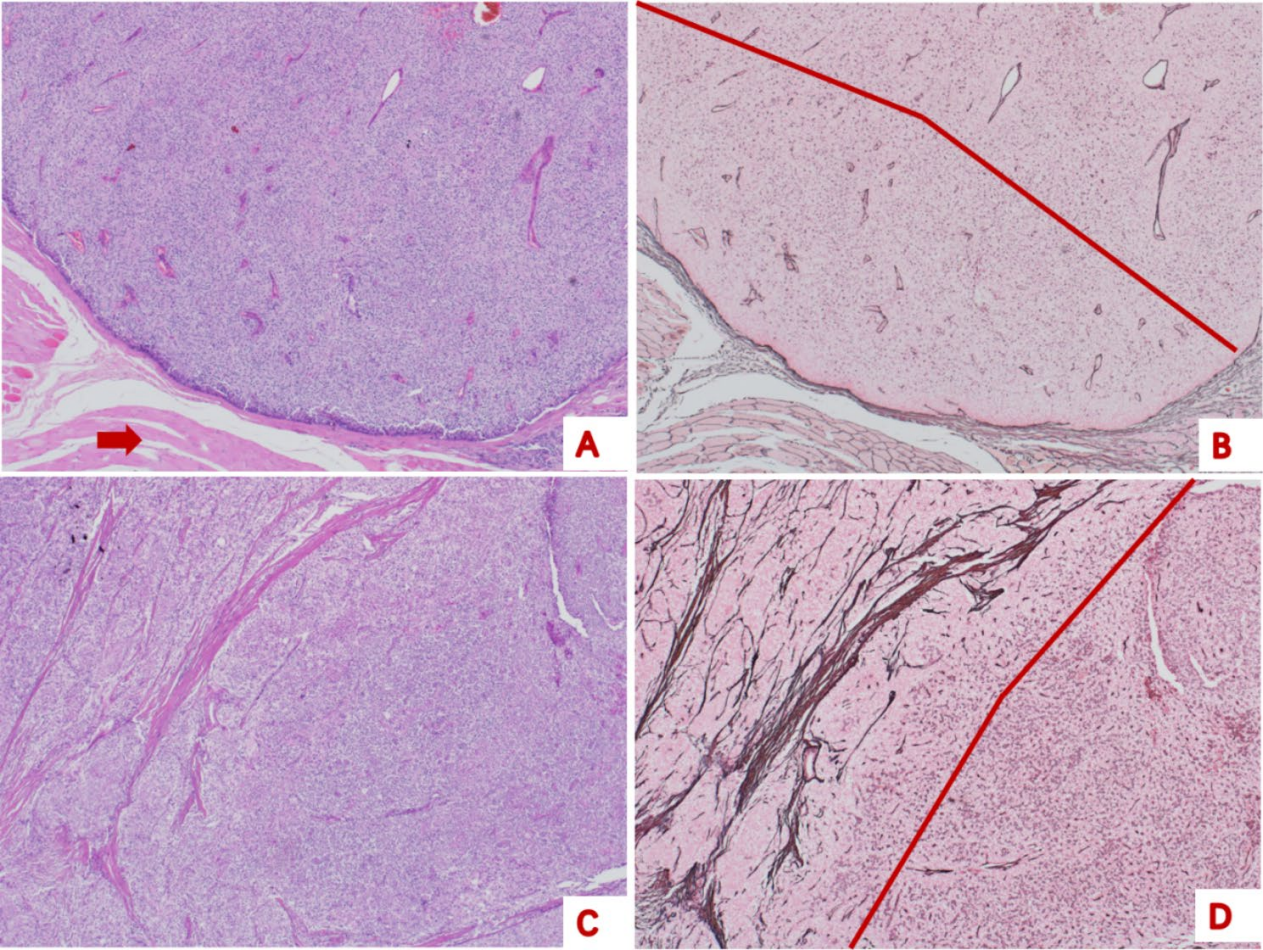
Representative histological features and reticular fibre staining of parathyroid carcinoma **A**, the tumour invaded the surrounding striated muscle tissue (arrow), and was diagnosed as a parathyroid carcinoma (haematoxylin eosin, 40×). **B**, a traversable field line can be seen, indicating the destruction of reticular fibre structure (reticular fibre staining, 40×). A and C show different regions of the same case (haematoxylin eosin, 40×). **D**, The reticular fibre structure was damaged in some, but not in other regions (reticular fibre staining, 40×)

**Fig. 4:**
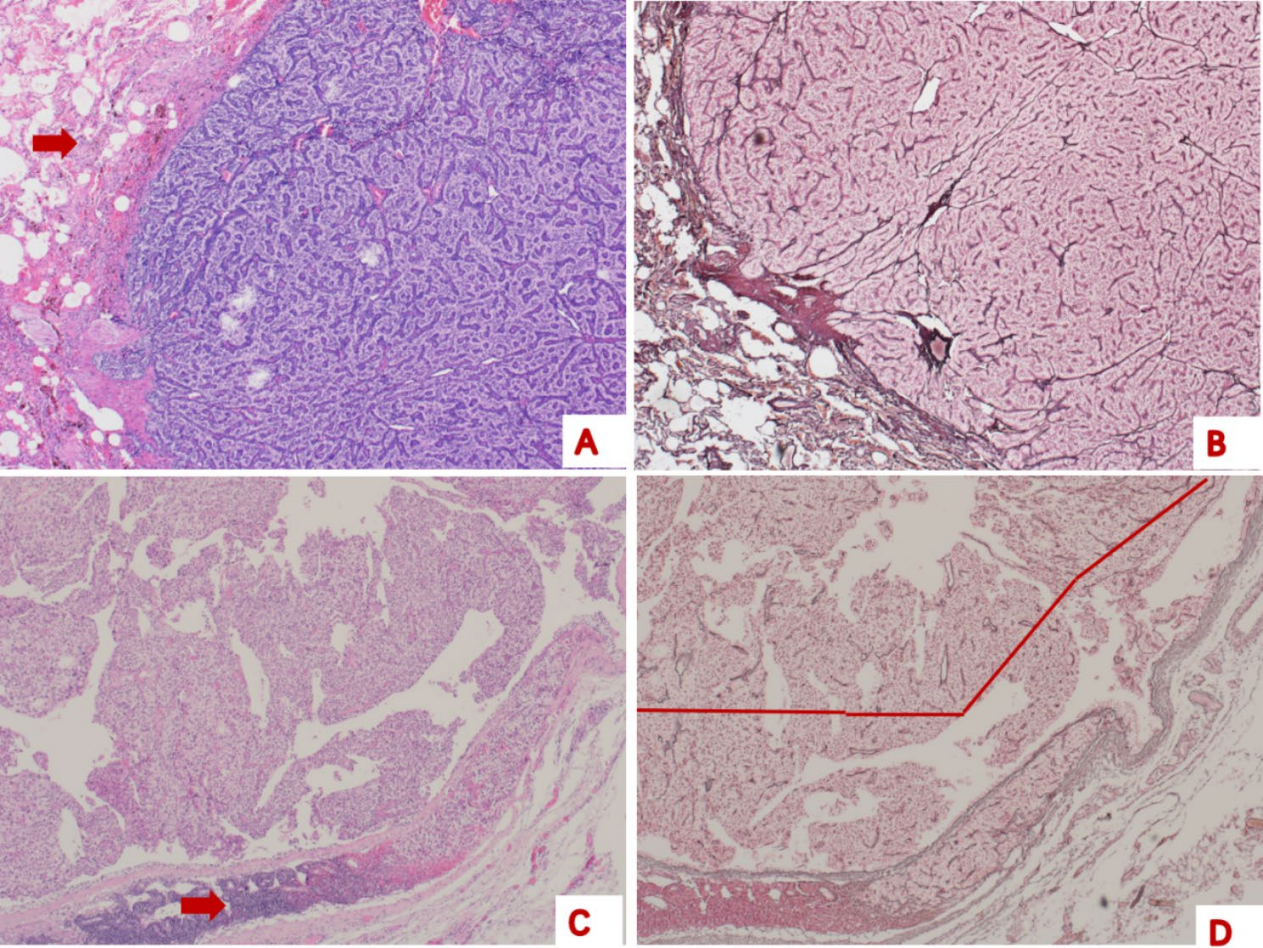
Representative histological features and reticular fibre staining of parathyroid carcinoma **A**, metastasis of parathyroid carcinoma to the lung. Normal lung tissue (arrow) can be seen (haematoxylin eosin, 40×). **B**, no destruction of the reticular fibre structure is visible (reticular fibre staining, 40×). **C**, metastasis of parathyroid carcinoma to the lymph node (arrow) (haematoxylin eosin, 40×). **D**, a traversable field line can be seen, indicating the destruction of reticular fibre structure (reticular fibre staining, 40×)

The destruction of RFS in the PTC group was more complete than that in the APT group (Figs. 3 and 4). The reticular fibres had disappeared completely or partially, with only a small amount remaining, with a sparse distribution, disorderly arrangement, and uneven thickness. Reticular fibrous tissue hyperplasia was observed in some cases, extending from the edge of the tumour cell nest to its centre.

### Incidence of RFS destruction in parathyroid tumours

Using the presence or absence of a traversable line as a criterion, this study further evaluated the incidence of RFS destruction in parathyroid tumours. None of the 50 PTA cases in this study showed evidence of RFS destruction. A difference in the incidence of RFS destruction between the APT and PTC groups was observed (P < 0.001, χ^2^-test), with values of 44.0% (11/25) and 86.1% (31/36), respectively. The sensitivity and specificity of RFS destruction in differentiating PTC from APT were 80.6% and 56.0%, respectively.

### Differences in the incidence of RFS destruction between primary PTC and recurrent and metastatic PTC

The PTC cases in this study included primary PTC and recurrent and metastatic PTC. We compared the difference in the incidence of RFS destruction between the two PTC groups. In the primary PTC group, the incidence of RFS destruction was 72.7% (8/11), whereas it was 92.0% (23/25) in the recurrent and metastatic PTC groups. No significant difference was found between these two PTC groups (P = 0.154, Fisher’s exact test). In this study, there was one case of primary PTC with cervical lymph node metastasis. Reticular fibre staining demonstrated the destruction of RFS in both the primary and metastatic lesions. Three patients with recurrent PTC had metachronous recurrence and metastasis during the follow-up period, involving seven lesions (four cases of recurrence, two cases of lymph node metastasis, and one case of lung metastasis). RFS destruction occurred in all seven lesions.

### Relationship between RFS destruction and clinicopathological features in APT and primary PTC

The relationship between RFS destruction and clinicopathological features of APT and primary PTC was evaluated. In the APT group, no significant correlation was found between RFS destruction and clinicopathological features (incomplete capsule invasion, fibre bands, obvious flake growth, large nucleolus, mitotic activity > 5/50 HPF, coagulation necrosis, and cytologic atypia) (P > 0.05, Fisher’s exact test). In the 11 cases in the primary PTC group, no significant correlation was found between RFS destruction and clinicopathological features (fibre bands, obvious flake growth, large nucleolus, mitotic activity > 5/50 HPF, coagulation necrosis, cytologic atypia, vascular invasion, lymphatic invasion, perineural invasion, invasion into adjacent anatomic structures, and documentation of metastatic disease (P > 0.05, Fisher’s exact test).

## Discussion

To date, there has been no previous studies on RFS characteristics in APT and PTC. In this report, we retrospectively studied 50 patients with PTA, 25 patients with APT, and 36 patients with PTC and observed the structural characteristics of reticular fibre staining. We found a delicate reticular fibre network structure in PTA, which was relatively complete despite differences in size and morphology. The RFS was discontinuous in a few areas and exhibited an irregular sinusoid structure. Kuşku et al. [4] performed reticular fibre staining on 97 parathyroid lesions in 85 patients. Their results showed short, thick fibres and anastomosing and nodular RFS in PTA. In both the APT and PTC groups in our study, incomplete areas of RFS were observed. The RFS partially disappeared in the APT group, whereas it disappeared more completely in the PTC group. Remnants of reticular fibres were sparse, disordered, and unevenly thickened. Reticular fibrous tissue hyperplasia was observed in a few patients with PTC, extending from the edge of the tumour cell nest to its centre; however, this feature was not found in the APT group. Mete et al. conducted reticular fibre staining in 50 cases of adrenocortical adenoma and found a mesh-like reticulin pattern surrounding individual cells in approximately 20% of the cases; these were further divided into complete, incomplete, and mixed specific patterns, of which the latter two patterns were dominant [5]. However, this was not observed in any lesions in the present study.

Currently, no diagnostic criteria for RFS destruction in parathyroid tumours exist, although such criteria have been described for other tumours. In the diagnosis of adrenal cortical tumours, RFS destruction is based on qualitative or quantitative criteria: quantitative alterations are characterized by various degrees of reticular fibre loss and disruption, whereas qualitative alterations involve an apparently conserved but irregular mesh composed of heterogeneous fibres with different thicknesses that surround small groups of cells or single cells [6, 7]. These qualitative or quantitative changes in the RFS can be defined as RFS destruction. In the diagnosis of pituitary adenoma, the appearance of the traversable line, mentioned in our report, is a qualitative indication of RFS destruction [8]. We observed a sparse RFS or even loss of the RFS in PTAs in this study, consistent with the findings of Kuşku et al. [4]. Although the RFS in this area is incomplete, it does not represent true destruction but may rather reflect slender reticular fibres or the spatial characteristics of the RFS. Therefore, in this study, the traversable line method was used to diagnose RFS destruction.

Using the above diagnostic criteria, we evaluated the incidence of RFS destruction in parathyroid tumours. Compared with PTA, APT and PTC had higher incidences of RFS destruction. The incidences of RFS destruction were 0%, 44%, and 86% in PTA, APT, and PTC cases, respectively, which was statistically significantly different. With morphological diagnosis considered as the gold standard for diagnosis, this feature had a high sensitivity (80.6%) for the diagnosis of PTC. Our data support the usefulness of observing RFS destruction in the differential diagnosis of PTC, which has not been reported to date. Reticular fibre staining may be a valuable tool for improving the diagnostic accuracy in parathyroid tumours. The destruction of the RFS may indicate that parathyroid tumours have unfavourable biological behaviours, suggesting the possibility of diagnosis of APT or PTC. However, this study found that the specificity of RFS destruction for PTC diagnosis was lower than expected (56%). In view of the low incidence of PTC and the fact that the histological diagnosis of PTC is currently controversial, a definite diagnosis requires higher sensitivity and specificity. Consequently, using RFS destruction as a diagnostic tool for invasive tumours is not completely satisfactory. Nonetheless, for cases with RFS destruction, the diagnosis is ultimately made only after complete submission of the tumour for assessment and extensive microscopic examination [9].

Reticulin fibre staining provides valuable support in confirming the diagnosis of adrenocortical carcinoma, and its assessment plays a fundamental role in the reticulin algorithm as a diagnostic tool [10]. RFS destruction is the basis of diagnosis of adrenocortical carcinoma. A diagnosis of adrenocortical carcinoma is rendered, if one of the following features is merged: > 5 mitoses/50 HPF, necrosis, and/or vascular invasion [11]. In addition, the reticulin algorithm demonstrated high interobserver reproducibility among pathologists from different institutions in diagnosis of adrenocortical carcinoma [10].

The five-year recurrence or metastasis rate of PTC is 40–82% [12]. Among the 36 PTC cases in this study, 19 were recurrent lesions and 6 were metastatic lesions. We found no significant difference in the RFS destruction score between primary and recurrent/metastatic PTCs (P = 0.154, Fisher’s exact test). Hence, we speculate that the primary and the recurrent/metastatic cases may have the same score for RFS destruction and that different lesions from the same patient have the same RFS. As the primary lesions in our recurrent or metastatic patients had been operated on in other hospitals, the characteristics of their primary lesions were unknown. Consequently, the relationship between RFS destruction and patient prognosis was unclear.

Clinicopathological characteristics indicate that appropriate areas should be selected for reticular fibre staining to improve the accuracy of diagnosis. Gill et al. studied parafibromin-negative cases and found that these cases demonstrated distinctive morphology, including extensive sheet-like, rather than acinar growth, eosinophilic cytoplasm, nuclear enlargement with distinctive coarse chromatin, perinuclear cytoplasmic clearing, a prominent arborizing vasculature, and, frequently, a thick capsule [13]. Parafibromin-negative has been considered as an abnormal finding that requires further molecular testing to confirm its biological relevance. We prioritized sections with diffuse/nodular/trabecular growth patterns of tumour tissue or sections in which the tumour cells were the densest. Our results showed that, in APT and primary PTC cases, RFS destruction was not related to the clinicopathological characteristics. Nevertheless, we found that in cases with fibrous bands, diffuse/normal/trabecular growth patterns had a higher proportion of RFS destruction.

In APT and PTC, the mechanism of RFS destruction is still unclear. The possible mechanism is the interaction between tumour cells and extracellular matrix. When PTA occurs, the interstitial tissue of parathyroid gland is capillary generation, forming a fine RFS. When PTC or APT is formed, the tumour cells proliferate excessively, the extracellular matrix and the expression of reticular fibres change, and the reticular fibre staining shows the destruction of RFS [8].

The most effective treatment for PTC is complete resection of all tumour tissue at the time of initial surgery. Therefore, accurate identification of PTCs before or during surgery helps with surgical planning and avoiding secondary salvage surgery. Previous studies have suggested that if the tumour can be felt, maximum tumour diameter exceeds 3 cm, total plasma calcium level exceeds 3.5 mmol/L, and blood PTH level is 10 times higher than the upper limit of the normal value, the tumour is likely to be a PTC [14]. As no presurgical technique is diagnostically definitive, assessment of PTC largely relies on the pathologist’s analysis of the surgical specimen. However, depending on the aforementioned clinical manifestations and biochemical indicators, it is difficult to determine the nature of parathyroid tumours before or during surgery. In fact, up to 86% of PTC cases are not recognized during surgery and are not fully resected [15]. Reticular fibre staining of intraoperative frozen specimens has been used for the diagnosis of pituitary adenoma. In 1977, Elasco et al. described the use of a rapid silver stain to diagnose adenomas on frozen sections; this stain is widely accepted as the diagnostic gold standard [16]. Noh et al. further improved the method, shortened the staining time, while maintaining similar accuracy as in the original method, and considered it a reliable diagnostic method [17]. Therefore, performing reticular fibre staining intraoperatively to determine whether RFS destruction is present can assist in the diagnosis of parathyroid tumours. We will further explore the value of improved reticular fibre staining technology for the intraoperative diagnosis of parathyroid tumours.

This study had a few limitations. First, this study used the diagnostic criteria for pituitary adenoma as the standard for the destruction of the RFS; however, these criteria may not be optimal for the diagnosis of parathyroid tumours. The diagnostic criteria need to be clearly defined and analysed in detail in the future. Second, the morphological characteristics of PTC complicated judging the destruction of RFS in a double-blind fashion, and to some extent, the results were subjective. Further rigorous, large-sample studies are needed to provide more information in the future.

In conclusion, this study described the RFS characteristics of parathyroid tumours, including PTA, APT, and PTC, which had not been described previously. We propose reticular fibre staining as a tool to improve the accuracy of parathyroid tumour diagnosis, and that the destruction of the RFS may indicate that parathyroid tumours have unfavourable biological behaviour.

## Data Availability

Is available upon request from the corresponding author.

## Abbreviations

APT: Atypical parathyroid tumour
HE: Haematoxylin and eosin
HPF: High-power field
PTA: Parathyroid adenoma
PTC: Parathyroid carcinoma
RFS: Reticular fibre structure
PTH: Parathyroid hormone
WHO: World Health Organisation

## References

[1] Erickson LA, Mete O, Juhlin CC, Perren A, Gill AJ (2022). Overview of the 2022 WHO classification of parathyroid tumors. Endocr Pathol.

[2] Cetani F, Marcocci C, Torregrossa L, Pardi E (2019). Atypical parathyroid adenomas: challenging lesions in the differential diagnosis of endocrine tumors. Endocr Relat Cancer.

[3] Juhlin CC, Nilsson IL, Lagerstedt-Robinson K, Stenman A, Bränström R, Tham E (2019). Parafibromin immunostainings of parathyroid tumors in clinical routine: a near-decade experience from a tertiary centre. Mod Pathol.

[4] Kuşku Çabuk F, Sar M, Canoğlu D, Dural C, Güneş ME (2020). Reticulin staining pattern in the differential diagnosis of benign parathyroid lesions. J Endocrinol Invest.

[5] Mete O, Gucer H, Kefeli M, Asa SL (2018). Diagnostic and prognostic biomarkers of adrenal cortical carcinoma. Am J Surg Pathol.

[6] Duregon E, Fassina A, Volante M, Nesi G, Santi R, Gatti G (2013). The reticulin algorithm for adrenocortical tumor diagnosis: a multicentric validation study on 245 unpublished cases. Am J Surg Pathol.

[7] Volante M, Bollito E, Sperone P, Tavaglione V, Daffara F, Porpiglia F (2009). Clinicopathological study of a series of 92 adrenocortical carcinomas: from a proposal of simplified diagnostic algorithm to prognostic stratification. Histopathology.

[8] Jarzembowski J, Lloyd R, McKeever P (2007). Type IV collagen immunostaining is a simple, reliable diagnostic tool for distinguishing between adenomatous and normal pituitary glands. Arch Pathol Lab Med.

[9] Kane BS, Sow M, Diagne N, Badji N, Seck M, Akpo G (2019). The parathyroid carcinoma: a diagnostic challenge before surgery. Tunis Med.

[10] Gambella A, Volante M, Papotti M (2023). Histopathologic features of adrenal cortical carcinoma. Adv Anat Pathol.

[11] Mete O, Erickson LA, Juhlin CC, de Krijger RR, Sasano H, Volante M (2022). Overview of the 2022 WHO classification of adrenal cortical tumors. Endocr Pathol.

[12] Zhu R, Wang Z, Hu Y (2020). Prognostic role of parafibromin staining and CDC73 mutation in patients with parathyroid carcinoma: a systematic review and meta-analysis based on individual patient data. Clin Endocrinol.

[13] Gill AJ, Lim G, Cheung VKY, Andrici J, Perry-Keene JL, Paik J (2019). Parafibromin-deficient (HPT-JT type, CDC73 mutated) parathyroid tumors demonstrate distinctive morphologic features. Am J Surg Pathol.

[14] Salcuni AS, Cetani F, Guarnieri V, Nicastro V, Romagnoli E, de Martino D (2018). Parathyroid carcinoma. Best Pract Res Clin Endocrinol Metab.

[15] Tan MH, Morrison C, Wang P, Yang X, Haven CJ, Zhang C (2004). Loss of parafibromin immunoreactivity is a distinguishing feature of parathyroid carcinoma. Clin Cancer Res.

[16] Velasco ME, Sindely SO, Roessmann U (1977). Reticulum stain for frozen-section diagnosis of pituitary adenomas. Technical note. J Neurosurg.

[17] Noh S, Kim SH, Cho NH, Kim SH (2015). Rapid reticulin fiber staining method is helpful for the diagnosis of pituitary adenoma in frozen section. Endocr Pathol.

